# Identification of Exosomal microRNAs and Their Targets in Papillary Thyroid Cancer Cells

**DOI:** 10.3390/biomedicines10050961

**Published:** 2022-04-21

**Authors:** Valentina Maggisano, Francesca Capriglione, Antonella Verrienti, Marilena Celano, Agnese Gagliardi, Stefania Bulotta, Marialuisa Sponziello, Catia Mio, Valeria Pecce, Cosimo Durante, Giuseppe Damante, Diego Russo

**Affiliations:** 1 Department of Health Sciences, University “Magna Graecia” of Catanzaro, 88100 Catanzaro, Italy; vmaggisano@unicz.it (V.M.); francesca.capriglione@studenti.unicz.it (F.C.); celano@unicz.it (M.C.); gagliardi@unicz.it (A.G.); bulotta@unicz.it (S.B.); 2 Department of Translational and Precision Medicine, “Sapienza” University of Rome, 00161 Rome, Italy; antonella.verrienti@uniroma1.it (A.V.); marialuisa.sponziello@uniroma1.it (M.S.); valeria.pecce@uniroma1.it (V.P.); cosimo.durante@uniroma1.it (C.D.); 3 Institute of Medical Genetics, Academic Hospital of Udine, Azienda Sanitaria Universitaria Integrata di Udine, 33100 Udine, Italy; catia.mio@uniud.it (C.M.); giuseppe.damante@uniud.it (G.D.)

**Keywords:** exosomes, thyroid cancer cells, miRNAs

## Abstract

The release of molecules in exosomal cargoes is involved in tumor development and progression. We compared the profiles of exosomal microRNAs released by two thyroid cancer cell lines (TPC-1 and K1) with that of non-tumorigenic thyroid cells (Nthy-ori-3-1), and we explored the network of miRNA–target interaction. After extraction and characterization of exosomes, expression levels of microRNAs were investigated using custom TaqMan Advanced array cards, and compared with those expressed in the total cell extracts. The functional enrichment and network-based analysis of the miRNAs’ targets was also performed. Five microRNAs (miR-21-5p, miR-31-5p, miR-221-3p, miR-222-3p, and let-7i-3p) were significantly deregulated in the exosomes of tumor cells vs. non-tumorigenic cells, and three of them (miR-31-5p, miR-222-3p, and let-7i-3p) in the more aggressive K1 compared to TPC-1 cells. The network analysis of the five miRNAs identified some genes as targets of more than one miRNAs. These findings permitted the identification of exosomal microRNAs secreted by aggressive PTC cells, and indicated that their main targets are regulators of the tumor microenvironment. A deeper analysis of the functional role of the targets of exosomal miRNAs will provide further information on novel targets of molecular treatments for these neoplasms.

## 1. Introduction

MicroRNAs (miRNAs) are small non-coding RNA molecules with a length of 21–23 nucleotides, able to regulate the expression of several genes [1], and their role in the evolution and progression of cancer has been demonstrated in many human diseases [1].

In recent years, many papers have reported the usefulness of miRNAs as potential biomarkers for the diagnosis and the follow-up of many tumors, including thyroid cancers [2,3]. In this regard, analysis of miRNAs expressed in thyroid tumor tissues [4,5] and investigation of circulating miRNAs [6] have been proposed as useful tools for the diagnosis and management of thyroid cancer patients. In particular, miR-146b-5p, miR-21, miR-221, and miR-222 have been proposed as biomarkers of papillary thyroid cancer (PTC), and their overexpression has been found to be associated with lymph node and distant metastases [7,8,9]. Moreover, there are also circulating miRNAs described as PTC biomarkers usable to discriminate patients and healthy control subjects. Among them, miR-222-3p, miR-221-3p, and miR-146a-5p have been reported in various papers [6,10,11,12,13,14,15], and miR-222 in particular might also be useful in discriminating PTC from nodular goiter patients [11], while miR-222 and miR-146 seem to be useful prognostic biomarkers to identify the patients with poorer outcomes [16].

Recently, the interest in miRNAs carried by exosomes has greatly increased. Exosomes are endosome-derived vesicles acting as cargoes of various cellular products, including proteins, nucleic acids, and lipids, and are involved in many functions, such as antigen presentation, intercellular communication, tissue homeostasis, and immunity [17]. In addition, an important role of exosomes has been proposed in the development, progression, and dissemination of cancer [18]. The presence of miRNAs as functional effectors contained in exosomes secreted by tumor cells has encouraged the study of exosomal miRNAs as potential biomarkers for the diagnosis and the follow-up of many neoplasms [2,3]. The research on the role of exosomal miRNAs in thyroid carcinoma has been focused mainly on their potential use as diagnostic or prognostic biomarkers [13,19,20,21,22] to identify the patients with a poor prognosis and high risk of disease recurrence [20,23,24,25]. However, these studies’ results are discordant, due to their pre-analytic and analytic variability—including the selection and collection of samples, as well as their methodological approaches—and the cited works have not shed light on the potential role of exosomal miRNAs as modulators of the functional activities of cancer cells.

In the present study, we analyzed the profiles of exosomal miRNAs secreted by two PTC cell lines (TPC-1, and the more aggressive K1), and compared them with that of non-tumorigenic thyroid cells (Nthy-ori-3-1). The difference in the expression of some miRNAs was also investigated between the two PTC cell lines with different behavior in terms of aggressiveness. Moreover, to shed light on the potential pathogenic role of the identified exosomal miRNAs secreted by PTC cells, we performed functional enrichment analysis of the miRNAs’ targets, and explored the network of miRNA–target interaction.

## 2. Materials and Methods

### 2.1. Cell Cultures

In this study we used two human PTC cell lines—TPC-1 and K1—chosen for their different behavior in terms of growth and invasiveness, with the K1 cells considered to be an in vitro model of aggressive PTC. As a control, we used the human non-tumorigenic thyroid cell line Nthy-ori-3-1, widely adopted as a model of normal human thyroid cells [26]. Cells were cultured in DMEM or RPMI (Thermo Fisher Scientific Inc., Waltham, MA, USA) media at 37 °C in a humidified 5% CO_2_ atmosphere, as previously described [27]. To confirm the identity of the cell lines, short tandem repeat analysis was performed by using the AmpFLSTR NGM SElect PCR Amplification Kit (Thermo Fisher Scientific Inc.) (Supplementary Table S1 [28]).

### 2.2. Exosome Extraction

Cells were seeded in 75 cm^2^ culture flasks at a density of 5 × 10^6^ (Nthy-ori-3-1), 4 × 106 (TPC-1) or 3.5 × 10^6^ (K1). The next day, the growth medium was replaced with fresh medium supplemented with 10% exosome-depleted fetal bovine serum (FBS, Thermo Fisher Scientific Inc.) for 48 h. The conditioned medium was harvested, and then centrifuged at 4000 rpm for 30 min to remove cellular debris. Then, exosomes were precipitated overnight at 4 °C with ExoQuick-TC (Systems Bioscience, Palo Alto, CA, USA), according to the manufacturer’s instructions. The pellets were resuspended in phosphate-buffered saline (PBS) and stored at −80 °C until use.

### 2.3. Exosome Characterization

#### 2.3.1. Dynamic Light Scattering (DLS)

Purified exosomes were diluted 1:25 in PBS, and their size distribution and polydispersity index were analyzed using a Zetasizer Nano ZS system (Malvern Instruments, Malvern, UK) [29]. Three measurements were taken for each aliquot.

#### 2.3.2. Protein Extraction and Western Blot

Exosomes and cells were lysed as previously described [6]. Thirty micrograms of each total protein extract were run on a 12% SDS–PAGE gel, transferred to PVDF membranes, blocked with T-PBS/milk (Triton 0.1%, PBS, and 5% non-fat dry milk), and incubated overnight with affinity-purified anti-CD63 (Systems Bioscience) and anti-calregulin (Santa Cruz Biotechnology Inc. Dallas, TX, USA) antibodies diluted 1:1000 and 1:500, respectively. Then, the membranes were washed in T-PBS and incubated with horseradish peroxidase-conjugated anti-rabbit or anti-mouse antibody (Transduction Laboratories, Lexington, KY, USA) in T-PBS/milk, diluted 1:20,000 or 1:5000, respectively. The protein was visualized by chemiluminescence using the Western blot detection system ECL Plus (Perkin Elmer, Monza, Italy).

### 2.4. RNA Extraction

Total RNA was isolated from thyroid cell culture samples using TRIzol reagent (Thermo Fisher Scientific Inc.), following the manufacturer’s protocol [30], and from purified exosomes isolated from conditioned media of thyroid cancer cells by using the Total Exosome RNA and Protein Isolation Kit (Thermo Fisher Scientific Inc.). Briefly, exosome pellets were dissolved in PBS and, in the final step, total RNA was eluted from the spin column membrane with 20 μL of DNase/RNase-free water. RNAs were quantified with a NanoDrop 2000 (Thermo Fisher Scientific Inc.).

### 2.5. cDNA Synthesis and miRNA Expression

For miRNA analysis, 10 ng of RNA from cells and from exosomes isolated in the media of cultured cells were reverse-transcribed using the TaqMan Advanced miRNA cDNA Synthesis Kit (Thermo Fisher Scientific Inc.), following the manufacturer’s instructions. Briefly, a poly(A) tail was added to one end and an adapter to the other end of each miRNA. Then, cDNA was synthesized using a universal RT primer that anneals to the poly(A) tail. Each cDNA was pre-amplified for 14 cycles. and 10-fold-diluted cDNA was used to perform real-time PCR. Expression levels of exosomal miRNAs were investigated using custom-designed TaqMan Advanced miRNA array cards, described as TaqMan low-density arrays (TLDAs) (Thermo Fisher Scientific Inc.), which analyzed a total of 48 miRNAs, including an endogenous and an exogenous control (miR-16 and cel-miR-39-3p, respectively). The other 46 miRNAs, reported in Supplementary Table S2, were chosen using three criteria: (1) the most deregulated miRNAs in PTC tissue samples of patients with vs. without lymph node metastasis; (2) miRNAs deregulated in PTC vs. normal thyroid tissue; and (3) circulating miRNAs, free or encapsulated in vesicles, detected in the serum/plasma of PTC patients, as reported by Capriglione et al. [22]. Microfluidic cards were set up as previously described [31], and run on a 7900HT Fast Real-Time PCR System (Thermo Fisher Scientific Inc.). Expression Suite software, (version 1.0.3, Thermo Fisher Scientific, Inc.) was used to calculate cycle threshold (Ct) values (cutoff, 35). Expression levels of the selected miRNAs were analyzed in total cellular extracts using specific TaqMan Advanced MicroRNA Assays (Thermo Fisher Scientific Inc.). Data were normalized using miR-16 as an endogenous control, and results were expressed using the 2^−ΔΔCt^ method [32]. Nthy-ori-3-1 extracts were used as calibrator samples.

### 2.6. Network Analysis and Enrichment Analysis of miRNA Targets

We used the MicroRNA Enrichment Turned Network (MIENTURNET, available at http://userver.bio.uniroma1.it/apps/mienturnet/, accessed on 31 January 2022). This tool, receiving a list of miRNAs as input, infers evidence of experimentally validated miRNA–target interactions (miRTarBase), and builds the network among them. In order to build a robust network, we selected only data from strong experimental methods. Moreover, MIENTURNET allowed us to perform the functional enrichment analysis of the miRNA target genes using the WikiPathways database [33].

### 2.7. Statistical Analysis

GraphPad Prism software, version 9.0 (GraphPad Software Inc., San Diego, CA, USA), was used for all statistical analysis. One-way ANOVA with Tukey’s multiple comparisons test was used to analyze miRNAs’ expression levels. All results are expressed as the mean ± standard deviation (SD), and were considered statistically significant at *p*-values lower than 0.05.

## 3. Results

### 3.1. Isolation and Characterization of Exosomes Derived from Thyroid Cells in Culture

**Figure 1:**
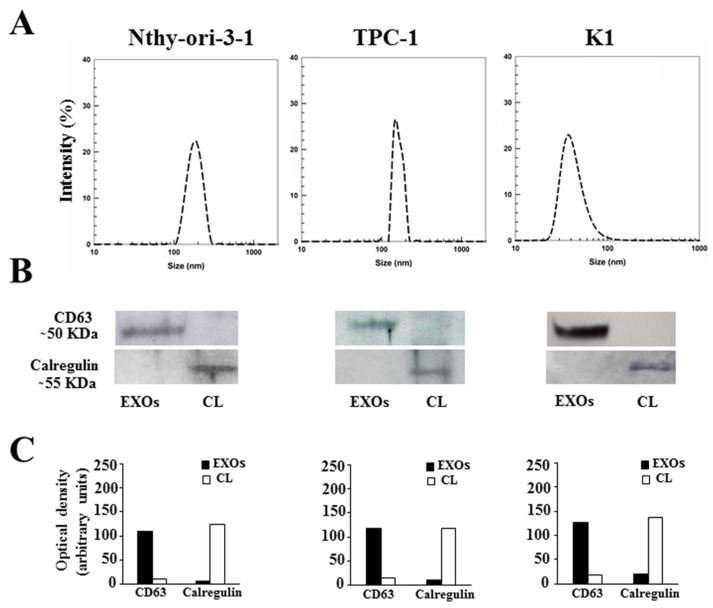
Characterization of Nthy-ori-3-1-, TPC-1-, and K1-cell-derived exosomes: (**A**) Mean sizes of exosomes extracted from conditioned media of Nthy-ori-3-1, TPC-1, and K1 cells. (**B**) Western blotting analysis of CD63 and calregulin in exosomal extracts (EXOs) and whole-cell lysate (CL) of Nthy-ori-3-1, TPC-1, and K1 cells. (**C**) Quantitative results of optical density of CD63 and calregulin.

First, we isolated exosomes from conditioned media of Nthy-ori-3-1, TPC-1, and K1 cells maintained for 48 h in exosome-depleted FBS to avoid contamination of the vesicles with FBS. Dynamic light scattering and Western blotting analysis were used to characterize the exosomes. As shown in Figure 1A, purified particles had a size distribution in the expected range of 40–160 nm. In addition, immunoblotting assays revealed the expression of CD63—a classical hallmark of exosomes [34]—in the exosomal extracts of all three cell lines (Figure 1B,C). We also evaluated the expression of the calregulin protein to exclude cell contamination in the exosome lysates. As shown in Figure 1B,C, the expression of this marker was detected in the cells, but not in the exosome extracts.

### 3.2. Expression Analysis of Exosomal miRNAs

**Figure 2:**
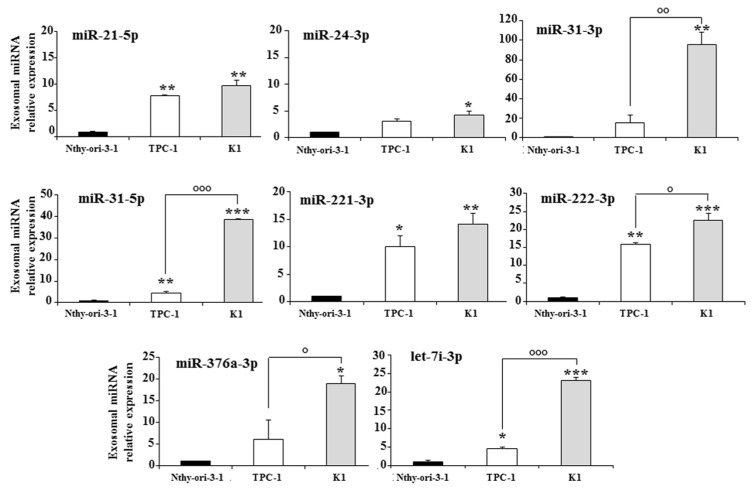
Exosomal miRNAs differentially expressed in the media of a non-tumorigenic thyroid cell line (Nthy-ori-3-1) and of two PTC cell lines (TPC-1 and K1); miRNA expression levels are reported as the mean expression value of each PTC cell line and normalized to the mean expression of the Nthy-ori-3-1 cell line (equal to 1). Error bars represent standard deviation; *p*-values were obtained by using one-way ANOVA with Tukey’s multiple comparison test: * *p* < 0.05, ** *p* < 0.01, *** *p* < 0.001 vs. Nthy-ori-3-1; ° *p* < 0.05, °° *p* < 0.01, °°° *p* < 0.001 vs. TPC-1.

Real-time-PCR-based TLDAs were used to detect miRNA levels in the exosomes present in culture media of Nthy-ori-3-1, TPC-1, and K1 cells. Analysis of exosomal miRNA expression revealed eight miRNAs deregulated in the tumor cell lines compared to the normal cells. Five miRNAs (miR-21-5p, miR-31-5p, miR-221-3p, miR-222-3p, and let-7i-3p) were significantly upregulated in both tumor cell lines compared to the non-tumorigenic cell line; three of them (miR-31-5p, miR-222-3p, and let-7i-3p) also showed a significant upregulation in the more aggressive K1 cells compared to the TPC-1 cells (Figure 2). The other three miRNAs (miR-24-3p, miR-31-3p, and miR-376a-3p) were upregulated only in K1 cells compared to the other cell lines.

### 3.3. Expression Analysis of the miRNAs in Total Cellular Extracts

**Figure 3:**
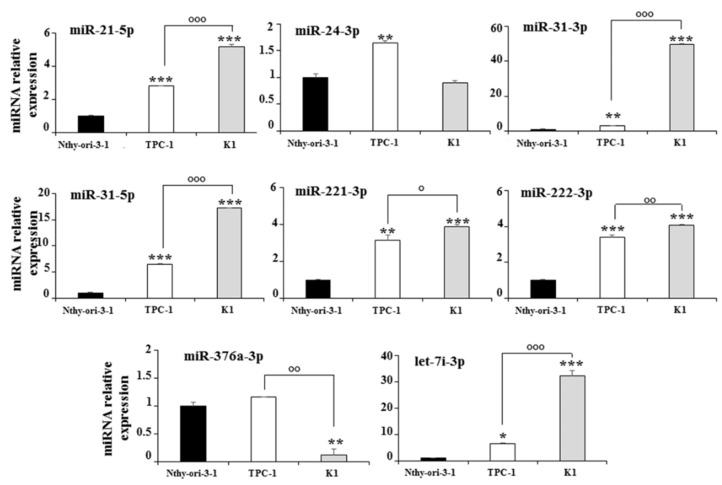
Expression levels of cellular miRNAs in Nthy-ori-3-1, TPC-1, and K1 cells; miRNA expression levels are reported as the mean expression value of each PTC cell line and normalized to the mean expression of the Nthy-ori-3-1 cells (equal to 1). Error bars represent standard deviation; *p*-values were obtained by using one-way ANOVA with Tukey’s multiple comparison test: * *p* < 0.05, ** *p* < 0.01, *** *p* < 0.001 vs. Nthy-ori-3-1; ° *p* < 0.05, °° *p* < 0.01, °°° *p* < 0.001 vs. TPC-1.

The expression of the eight deregulated exosomal miRNAs was also analyzed in the total cellular extracts. Six miRNAs (miR-21-5p, miR-31-3p, miR-31-5p, miR-221-3p, miR-222-3p, and let-7i-3p), including the three upregulated in K1 vs. TPC-1 cells, showed a similar behavior in the cell and exosomal compartments, while miR-376a-3p and miR-24-3p were significantly downregulated in the total extracts of K1 and upregulated in those of TPC-1 cells (Figure 3).

### 3.4. Functional Enrichment and Network-Based Analysis of Exosomal miRNAs Target Genes

**Figure 4:**
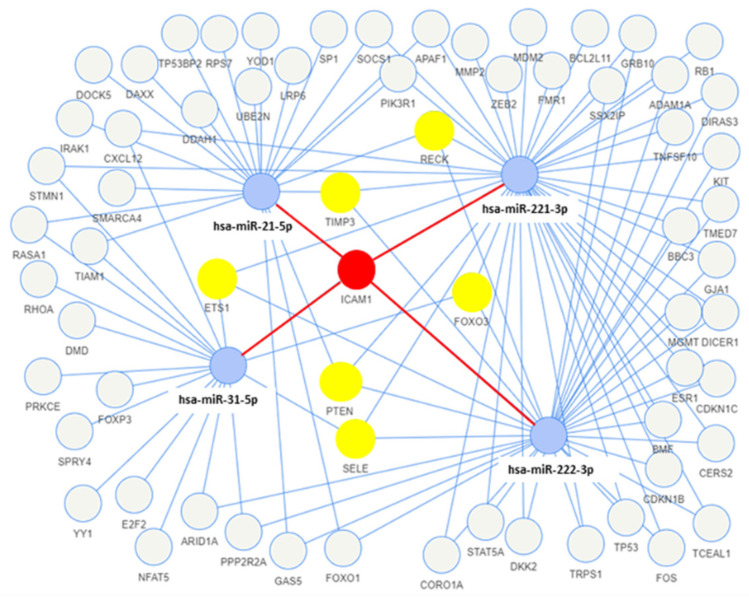
Network of the miRNA–target interactions using miRTarBase, with strong experimental evidence (e.g., luciferase assay, Western blotting). Blue circles are miRNAs, yellow circles are the target genes of three miRNAs, and the red circle is the target gene of four miRNAs.

**Figure 5:**
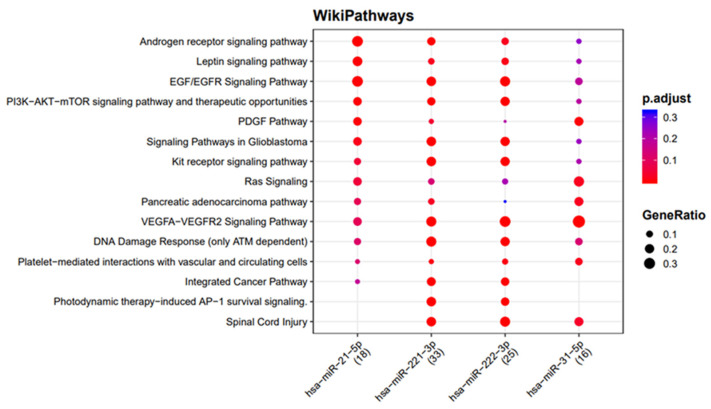
Functional enrichment of miRNA target genes using the WikiPathways database. The colors of the circles represent the adjusted *p*-values (FDR), whereas their size represents the number of miRNA targets found to be annotated in each category over the total number of recognized targets (indicated in round brackets).

The network analysis was used to identify the genes targeted by more than one miRNA found to be upregulated in the exosomes of both tumor cell lines. Among the target genes, seven (*ICAM1*, *TIMP3*, *RECK*, *PTEN*, *FOXO3*, *SELE*, and *ETS1*) were the target of more than two exosomal miRNAs, and one of them (*ICAM1*) was found to interact with four miRNAs (miR-31-5p, miR-221-3p, miR-21-5p, and miR-222-3p) (Figure 4). Moreover, the functional enrichment of miRNA target genes showed that the target genes are involved in many pathways, and they are mainly involved in the VEGFA-VEGFR2 and EGF-EGFR signaling pathways and the ATM-dependent DNA damage response (Figure 5).

## 4. Discussion

Identification of novel biomarkers and therapeutic targets to exploit more efficient options for the treatment of more aggressive PTCs is still an open challenge [35,36]. In this regard, particular interest has been devoted to the study of circulating miRNAs [6,37]. However, the short half-life of plasmatic miRNAs is a limit for early detection in the sera of the patients, which may be overcome if the search is focused on those protected by the enzymatic degradation through the entrapment in vesicular exosomes. Indeed, exosomes show all of the requisites of circulating cargoes optimal for containing small RNA molecules [23]. In addition, little information is available on the role of exosomal miRNAs secreted by thyroid cancer cells.

In the present work, analysis of the exosomal miRNAs secreted by two PTC cells, along with the comparison with those released by non-tumorigenic thyroid cells, allowed the identification of a few exosomal miRNAs (miR-21-5p, miR-31-5p, miR-221-3p, miR-222-3p, and let-7i-3p) deregulated in thyroid tumor cells, and three of them (miR-31-5p, miR-222-3p, and let-7i-3p) appeared to be specific to the K1 cells—a PTC cell line with more aggressive behavior in terms of growth rate and invasiveness. Moreover, comparison of the expression levels of the exosomal and cellular miRNAs in the cancer cells revealed a strong correlation between the two compartments for most of the deregulated miRNAs, suggesting an involvement of these miRNAs in thyroid carcinogenesis as well. These results are in accordance with published data on the upregulation of most of these miRNAs in PTC [5,38,39,40], while no data are available for let-7i-3p.

In particular, recent data reported that, in PTC, miR-221-3p can promote proliferation and invasion by targeting *TIMP3* [41], and inhibits apoptosis by suppressing FOXP2 expression through activation of the Hedgehog pathway [42]. miR-222-3p has been shown to enhance proliferation, migration, and invasion of PTC by downregulating *PTEN* expression and activating the AKT signaling pathway [43,44]. The same effect on the PTEN/AKT pathway has been demonstrated by miR-21-5p in PTC cells, through which the miRNA induces cell proliferation and inhibits apoptosis [45,46].

Moreover, the overexpression of the miRNAs 221-3p and 222-3p may be responsible for radioresistance, probably through the activation of the STAT3 signaling pathway [47]

With respect to the circulating let-7 family in PTC, data in the literature are limited. Perdas et al. found let-7a, let-7c, let-7d, let-7f, and let-7i to be upregulated in the plasma of PTC patients, but for let-7i the difference was not statistically significant [48]. However, to confirm these results, a larger group of patients is needed.

In addition, a variety of evidence supports the role of some of these exosomal miRNAs in the crosstalk between tumor cells and non-malignant cells present in the tumor microenvironment (TME). Indeed, it has been shown that PTC cells, under hypoxic conditions, communicate with endothelial cells through exosome miR-21-5p to induce tumor angiogenesis [49], and a recent meta-analysis has confirmed the role of miR-222 as biomarker of poor prognosis in terms of both overall survival and secondary outcomes in PTC [50].

An emerging concept in the study of the functional effects of the miRNAs identified in preclinical experimental models is the need to identify their targets, and since different miRNAs may interfere with the regulation of the expression of identical genes, we used the network analysis of miRNA–target interactions to search for targets in common among the selected miRNAs, and used miRNA–target enrichment analysis to shed more light on the role of the identified exosomal miRNAs secreted by PTC cancer cells. These analyses revealed that the most affected cellular processes are the VEGFA−VEGFR2 and PDGF signaling pathways, the ATM-dependent DNA damage response, and the PI3K−AKT−mTOR signaling pathway, all of which are known to be involved in thyroid tumorigenesis [35].

Interestingly, for the first time, the present results suggest that some specific miRNAs are expressed by thyroid cancer cells, and can act at a distance using an exosome-dependent communication system. Accordingly, a role of exosomes as modulators of the cancer–TME communication by transferring miRNAs to recipient cells has also been proposed, and is under investigation in many neoplasms [14].

In the present study, the network analysis of miRNA–target interactions allowed us to identify seven genes (*ICAM1*, *FOXO3*, *SELE*, *ETS1*, *RECK*, *PTEN,* and *TIMP3*) as common targets of at least three miRNAs. All of these genes codify proteins involved in the modulation of angiogenesis and immune response to cancer.

In particular, the *ICAM1* and *SELE* genes encode for two adhesion molecules whose expression has been reported to be increased in PTC [51,52]. *PTEN* is one of the most important regulators of PI3K signaling, and its dysregulation has dramatic effects on this pathway. Deletion of the *PTEN* gene has been demonstrated to be sufficient for the initiation of thyroid cancers in vivo [53]. *FOXO3* plays a tumor-suppressor role in differentiated thyroid cancers where its expression is downregulated [54]; notably, FOXOs seem to promote the antitumor immune response by negatively regulating several immunosuppressive factors (e.g., PD-L1 and VEGF) and positively regulating chemokine attractants [55]. *ETS1* plays several critical roles in immunity and angiogenesis, and it has been found to be upregulated in PTC tissues, where it inhibits apoptosis [56,57,58]. The *TIMP3* and *RECK* genes encode for proteins that inhibit matrix metalloproteinase, and have been demonstrated to play a tumor-suppressor role by inhibiting angiogenesis, invasion, and metastasis [59].

Altogether, these findings suggest that the tumor microenvironment may be the main target of the exosomal miRNAs secreted by thyroid tumor cells. Investigation of the expression and roles of these miRNA targets in all subtypes of thyroid cancer will enable the provision of additional information for better characterizing the in vivo effects of these miRNAs, as well as the identification of novel molecular targets useful for more appropriate treatment of thyroid cancer patients.

## 5. Conclusions

In conclusion, we found that thyroid cancer cells release exosomes containing miRNAs that may allow the tumor cells to interact both with one another and with cells of the TME, contributing to an aggressive phenotype by favoring invasion, angiogenesis, and other metastatic properties.

## Data Availability

Not applicable.

## Supplementary Materials

The following are available online at https://www.mdpi.com/article/10.3390/biomedicines10050961/s1, Table S1: STR analysis of utilized cell lines; Table S2: miRNAs in TaqMan Advanced miRNA Array Cards. Reference [28] is cited in the supplementary materials.
