# Supplementary material for: Identification of Exosomal microRNAs and Their Targets in Papillary Thyroid Cancer Cells
Source: Biomedicines. 2022 Apr 21;10(5):961. doi: 10.3390/biomedicines10050961 (PMC9138952; doi:10.3390/biomedicines10050961)
Supplement: Supplementary file 1 [file biomedicines-10-00961-s001.zip › biomedicines-1638727-supplementary.pdf]

Supplementary Materials

Table S1. STR analysis of utilized cell lines.

	Cell Lines		
**STR**	**K1**	**TPC-1**	**Nthy-ori-3-1**
D2S1338	20/23	ND	ND
D3S1358	18	16/17	14/16
D8S1179	15	11/17	12
D18S51	18	13/16	14
D21S11	30/31.2	30/31.2	29/30
FGA	21/24	20/21	21/22
TH01	6/9	9	ND
vWA	ND	14/18	16/18
D16S539	ND	ND	12/13

Abbreviations: ND, Not Done; STR, short tandem repeat. Numbers indicate the identified alleles. All alleles of the three lines are identical to those reported in Cellosaurus (Expasy) (https://web.expasy.org/cellosaurus/, accessed on 28 March 2022) [28]. STRs have been evaluated by using the AmpFlSTR NGM Select PCR amplification Kit.

Table S2. miRNAs in TaqMan Advanced miRNA Array Cards.

miRNAs	
hsa-miR-16-5p	hsa-miR-337-3p
hsa-miR-19b-15p	hsa-miR-375-3p
hsa-miR-21-5p	hsa-miR-376a-3p
hsa-miR-24-2-5p	hsa-miR-382-5p
hsa-miR-24-3p	hsa-miR-485-3p
hsa-miR-31-3p	hsa-miR-506-3p
hsa-miR-31-5p	hsa-miR-508-3p
cel-miR-39-3p	hsa-miR-508-5p
hsa-miR-127-3p	hsa-miR-509-3p
hsa-miR-127-5p	hsa-miR-514a-3p
hsa-miR-135b-3p	hsa-miR-551b-3p
hsa-miR-136-3p	hsa-miR-675-3p
hsa-miR-136-5p	hsa-miR-758-3p
hsa-miR-146a-5p	hsa-miR-889-3p
hsa-miR-146b-3p	hsa-miR-934
hsa-miR-146b-5p	hsa-miR-944
hsa-miR-181a-5p	hsa-miR-3136-5p
hsa-miR-199b-5p	hsa-miR-4772-3p
hsa-miR-221-3p	hsa-miR-6842-3p
hsa-miR-222-3p	hsa-miR-6843-3p
hsa-miR-222-5p	hsa-miR-6854-5p
hsa-miR-223-3p	hsa-miR-6860
hsa-miR-329-3p	hsa-miR-7156-5p
hsa-miR-335-5p	hsa-let-7i-3p
